# Does training of general practitioners for intensive treatment of people with screen-detected diabetes have a spillover effect on mortality and cardiovascular morbidity in ‘at risk’ individuals with normoglycaemia? Results from the ADDITION-Denmark cluster-randomised controlled trial

**DOI:** 10.1007/s00125-017-4230-6

**Published:** 2017-03-09

**Authors:** Rebecca K. Simmons, Niels H. Bruun, Daniel R. Witte, Knut Borch-Johnsen, Marit E. Jørgensen, Annelli Sandbæk, Torsten Lauritzen

**Affiliations:** 1 MRC Epidemiology Unit, University of Cambridge School of Clinical Medicine, Cambridge, UK; 2 Department of Public Health, Aarhus University, Building 1260, Bartholins Allé 2, DK-8000 Aarhus C, Denmark; 3 Danish Diabetes Academy, Odense, Denmark; 4 Aarhus Institute of Advanced Studies, Aarhus University, Aarhus, Denmark; 5 Holbæk Hospital, Holbæk, Denmark; 6 Steno Diabetes Center Copenhagen, Gentofte, Denmark; 7 National Institute of Public Health, Southern Denmark University, Copenhagen, Denmark

**Keywords:** Cardiovascular disease, Diabetes, High risk, Intensive treatment, Normoglycaemic, Routine care, Screening, Spillover, Trial

## Abstract

**Aims/hypothesis:**

Within a trial of intensive treatment of people with screen-detected diabetes, we aimed to assess a potential spillover effect of the trial intervention on incident cardiovascular disease (CVD) and all-cause mortality among people who screened positive on a diabetes risk questionnaire but who were normoglycaemic.

**Methods:**

In the Anglo–Danish–Dutch Study of Intensive Treatment In People with Screen-Detected Diabetes in Primary Care (ADDITION)-Denmark trial, 175 general practices were cluster-randomised into: (1) screening plus routine care of individuals with screen-detected diabetes (control group); or (2) screening plus training and support in intensive multifactorial treatment of individuals with screen-detected diabetes (intervention group). We identified all individuals who screened positive on a diabetes risk questionnaire in ADDITION-Denmark but were normoglycaemic following biochemical testing for use in this secondary analysis. After a median 8.9 years follow-up, we used data from national registers to compare rates of first CVD events and all-cause mortality in individuals in the routine care group with those in the intensive treatment group.

**Results:**

In total, 21,513 individuals screened positive for high risk of diabetes but were normoglycaemic on biochemical testing in ADDITION-Denmark practices between 2001 and 2006 (10,289 in the routine care group and 11,224 in the intensive treatment group). During 9 years of follow-up, there were 3784 first CVD events and 1748 deaths. The incidence of CVD was lower among the intensive treatment group compared with the routine care group (HR 0.92 [95% CI 0.85, 0.99]). This association was stronger among individuals at highest CVD risk (heart SCORE ≥ 10; HR 0.85 [95% CI 0.75, 0.96]). There was no difference in mortality between the two treatment groups (HR 1.02 [95% CI 0.92, 1.14]).

**Conclusions/interpretation:**

Training of general practitioners to provide target-driven intensive management of blood glucose levels and other cardiovascular risk factors showed some evidence of a spillover effect on the risk of CVD over a 9 year period among individuals at high risk of diabetes. The effect was particularly pronounced among those at highest risk of CVD. There was no effect on mortality.

***Trial registration*:**

ClinicalTrials.gov NCT00237549

## Introduction

Screening for type 2 diabetes inevitably identifies more people at high risk of developing diabetes and cardiovascular disease (CVD) than those with undiagnosed prevalent disease. Little is known about these high risk individuals following their participation in a screening programme. While well-established guidelines for the treatment of diabetes exist, most countries do not specify how to treat individuals who screen positive following completion of a diabetes risk questionnaire but are normoglycaemic on biochemical testing.

In the Anglo–Danish–Dutch Study of Intensive Treatment In People with Screen-Detected Diabetes in Primary Care (ADDITION)-Denmark trial (ClinicalTrial.gov registration no. NCT00237549) [1], 175 general practices were cluster-randomised to routine care or to receive training and support in the implementation of an intensive treatment programme for individuals with screen-detected diabetes. This included lifestyle intervention and CVD risk factor management. The intervention was associated with a significant increase in redeemed cardioprotective medication and a non-significant 17% risk reduction in CVD events among individuals with screen-detected diabetes over 5 years of follow-up [2]. Given the favourable increase in cardioprotective medication observed in the intensive treatment practices, we wanted to investigate whether the education and guidelines we offered may have also had an impact on the management of patients with normal blood glucose levels following screening.

In order to assess a potential spillover effect of the trial intervention among practices taking part in ADDITION-Denmark, we compared rates of first CVD events and all-cause mortality among people who screened positive on the diabetes risk questionnaire but who were normoglycaemic on biochemical testing in the routine care (control) and intensive treatment trial groups.

## Methods

ADDITION-Denmark consists of two phases: (1) a stepwise screening programme; and (2) a cluster-randomised parallel-group trial comparing the effects of intensive multifactorial treatment with routine care among individuals with screen-detected type 2 diabetes [1, 2]. In brief, between 2001 and 2006, we performed a population-based stepwise screening programme among people aged 40–69 years without known diabetes in 175 general practices in Denmark. Eligible individuals were sent a diabetes risk score questionnaire [3] with an invitation to visit their family doctor for a diabetes test and a cardiovascular risk assessment (heart SCORE) [4] if they scored ≥ 5 points (maximum 15 points) on the risk questionnaire. The diabetes risk score questionnaire estimates diabetes risk using age, sex, BMI, known hypertension, leisure time physical activity and family history of diabetes [3]. The heart SCORE estimates fatal CVD risk using age, sex, smoking, systolic blood pressure and total cholesterol [4]. Participants who attended a screening appointment underwent measurement of height, weight, blood pressure, random blood glucose (RBG), total cholesterol and HbA_1c_. Individuals with an RBG ≥ 5.5 mmol/l or HbA_1c_ ≥ 5.8% (40 mmol/mol) were invited to return to the practice for further testing. The WHO 1999 criteria, based on a standard OGTT, were used to diagnose diabetes [5]. Participants diagnosed with type 2 diabetes were subsequently managed according to the treatment regimen to which their practice had been allocated: routine care (control) or intensive treatment. Ethical approval for the trial was granted by the Region Midt Ethical Committee, Denmark. As this was a registry-based study using anonymised data, participants did not give informed consent. This approach was approved by the Danish Data Protection Agency and the Danish Health and Medicine Authority.

For those diagnosed with diabetes, general practitioners and nurses received training and support in delivering intensive treatment via small group or practice-based educational meetings where treatment targets/algorithms, lifestyle advice and supporting evidence were discussed [2]. Intensive treatment practices received additional funding to support the delivery of care, which included target-driven management of hyperglycaemia and blood pressure and cholesterol levels by medical treatment and promotion of healthy lifestyles, based on the stepwise regimen used in the Steno-2 study and other trial results. In the routine care (control) group, general practitioners were advised to follow Danish national recommendations for diabetes treatment and received no further follow-up. In both groups, practitioners were encouraged to treat normoglycaemic individuals with a heart SCORE ≥ 5, according to Danish guidelines [6].

In this secondary analysis, in order to assess the potential spillover effect of the intervention, we identified individuals who underwent screening as part of ADDITION-Denmark and who were normoglycaemic on biochemical testing. Normoglycaemia in our study refers to individuals with an RBG < 5.5 mmol/l and HbA_1c_ ≤ 6% (42 mmol/mol) at the first visit/blood test, and people with fasting blood glucose < 5.6 mmol/l and 2 h blood glucose following an OGTT < 7.8 mmol/l. Participants were followed for a median of 8.9 years to 31 December 2011, when national registers were searched for information on vital status and a composite of first event of cardiovascular death (ICD-10 codes I60 to I69, I20 to I25, and I46), non-fatal ischaemic heart disease (ICD-10 codes I20 to I25, and I46) or non-fatal stroke (ICD-10 code I60 to I69).

### Statistical analysis

Characteristics were summarised separately in the intensive treatment and routine care (control) groups. Date of entry to the study was set as date of invitation to screening. Individuals were censored on the date of first event following invitation for screening (for the incident CVD analysis), upon death, or on the 31 December 2011 (final date of follow-up), depending on which occurred earliest. HRs comparing incident CVD events and all-cause mortality between the groups were estimated with a Cox proportional hazards regression model and we accounted for clustering at the general practitioner level. We tested the proportional hazards assumption by including a variable for treatment by time interaction in the Cox regression model (*p* > 0.05). We also examined these associations in all individuals with a heart SCORE of ≥ 0 to < 5, ≥5 to <10, and ≥ 10. All analyses were completed using Stata Version 14.1 (StataCorp, College Station, TX, USA).

## Results

Between 2001 and 2006, 21,513 individuals were found with normal blood glucose following screening in ADDITION-Denmark practices (10,289 in the routine care [control] group and 11,224 in the intensive treatment group). The groups had similar baseline characteristics, with similar proportions of men (51%) and numbers of individuals with >15 years education (26%) and previous CVD (∼4.5%; Table 1). The mean age in both groups was 59 years. The proportion of individuals redeeming lipid-lowering, glucose-lowering and anti-hypertensive medication was similar throughout follow-up (Table 1).

**Table 1:** Characteristics of normoglycaemic individuals following diabetes screening in the ADDITION-Denmark study (*n* = 21,513), by treatment group

	NGT		NGT and heart SCORE ≥ 0 to < 5		NGT and heart SCORE ≥ 5 to < 10		NGT and heart SCORE ≥ 10	
	Routine care group (*n* = 10,289)	Intensive treatment group (*n* = 11,224)	Routine care group (*n* = 4890)	Intensive treatment group (*n* = 5347)	Routine care group (*n* = 2450)	Intensive treatment group (*n* = 2603)	Routine care group (*n* = 1795)	Intensive treatment group (*n* = 1925)
Male sex, *n* (%)	5251 (51.0)	5749 (51.2)	1627 (33.3)	1745 (32.6)	1608 (65.6)	1705 (65.5)	1515 (84.4)	1625 (84.4)
Age (SD)	59.1 (6.9)	59.1 (7.0)	55.8 (6.4)	55.8 (6.4)	62.1 (5.1)	62.2 (4.8)	65.1 (4.0)	65.2 (4.1)
Education level, *n* (%)								
0–10 years	3191 (31.5)	3608 (32.6)	1453 (30.1)	1613 (30.5)	835 (34.6)	872 (34.0)	611 (34.7)	672 (35.5)
10–15 years	4297 (42.4)	4555 (41.1)	2075 (43.0)	2189 (41.4)	1005 (41.6)	1028 (40.0)	735 (41.8)	796 (42.1)
≥ 15 years	2640 (26.1)	2908 (26.3)	1297 (26.9)	1486 (28.1)	575 (23.8)	668 (26.0)	414 (23.5)	424 (22.4)
Previous CVD, *n* (%)	470 (4.6)	495 (4.4)	166 (3.4)	167 (3.1)	141 (5.8)	146 (5.6)	97 (5.4)	100 (5.2)
Redeemed anti-hypertensive medication, *n* (%)^a^								
Year 2000	2429 (23.6)	2663 (23.7)	1126 (23.0)	1199 (22.4)	608 (24.8)	667 (25.6)	457 (25.5)	506 (26.3)
Year 2005	4124 (40.7)	4383 (39.6)	1749 (36.1)	1814 (34.2)	1041 (43.2)	1100 (42.9)	904 (52.0)	977 (52.8)
Year 2010	5265 (54.3)	5692 (53.9)	2256 (47.6)	2411 (46.8)	1317 (58.0)	1406 (57.6)	1120 (70.8)	1198 (71.4)
Redeemed glucose-lowering medication, *n* (%)^a^								
Year 2000^b^								
Year 2005	21 (0.2)	21 (0.2)	9 (0.2)	12 (0.2)	5 (0.2)	^b^	6 (0.3)	5 (0.3)
Year 2010	156 (1.6)	184 (1.7)	74 (1.6)	86 (1.7)	34 (1.5)	43 (1.8)	39 (2.5)	33 (2.0)
Redeemed lipid-lowering medication, *n* (%)^a^								
Year 2000	383 (3.7)	374 (3.3)	138 (2.8)	135 (2.5)	125 (5.1)	112 (4.3)	86 (4.8)	100 (5.2)
Year 2005	1573 (15.5)	1692 (15.3)	602 (12.4)	638 (12.0)	452 (18.7)	487 (19.0)	369 (21.2)	401 (21.7)
Year 2010	3035 (31.3)	3238 (30.7)	1247 (26.3)	1341 (26.0)	779 (34.3)	845 (34.6)	652 (41.2)	685 (40.8)
HR for CVD (95% CI)	0.92 (0.85, 0.99)		0.90 (0.80, 1.02)		0.98 (0.84, 1.13)		0.85 (0.75, 0.96)	
HR for all-cause mortality (95% CI)	1.02 (0.92, 1.14)		1.07 (0.89, 1.30)		0.88 (0.75, 1.04)		1.06 (0.90, 1.26)	

Data are presented as *n* (%), mean (SD) or HR (95% CI)

Please note that numbers in the three heart SCORE groups do not add up to the total cohort size (*n* = 21,513) because of missing data for some individuals, which prevented us from computing the heart SCORE. Similarly, numbers for education do not add up to the total cohort size because of missing data

^a^Numbers for cardioprotective medication in years 2005 and 2010 were calculated using the individuals who were alive at the beginning of that year as the denominator

^b^Numbers for redeemed glucose-lowering medication are too small to report (Statistics Denmark regulations)

NGT, normal glucose tolerance

**Fig. 1:**
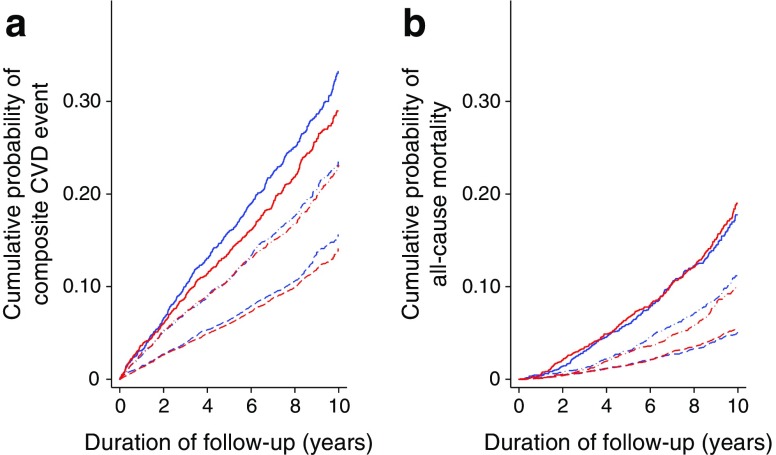
Cumulative incidence of (**a**) CVD and (**b**) all-cause mortality among individuals with normal glucose tolerance in the ADDITION-Denmark intensive treatment and routine care groups (2001–2011). This figure is unadjusted. Routine care group, blue; intensive treatment group, red. Heart SCORE ≥ 0 to <5, dashed line; heart SCORE ≥ 5 to <10, dot/dash line; heart SCORE ≥10, solid line

Median (interquartile range) duration of follow-up was 8.9 years (8.2–10.0). During follow-up, there were 1904 CVD events (17.0%) in the intensive treatment group and 1880 (18.3%) events in the routine care group. The incidence of CVD was lower among the intensive treatment group compared with the routine care group (HR 0.92 [95% CI 0.85, 0.99]; Table 1 and Fig. 1a). This association was more pronounced among individuals with a heart SCORE ≥10 (HR 0.85 [95% CI 0.75, 0.96]). There were 923 deaths (8.2%) in the intensive treatment group and 825 (8.0%) deaths in the routine care group. Of these, 125 (13.5%) and 106 (12.8%) were CVD-related deaths in the intensive treatment and routine care groups, respectively. The incidence of all-cause mortality was similar between the two treatment groups (HR 1.02 [95% CI 0.92, 1.14]; Table 1 and Fig. 1b) across all levels of CVD risk.

## Discussion

We found some evidence for a spillover effect from the intensive treatment of people with screen-detected diabetes to those with normal blood glucose levels. Among normoglycaemic individuals, the incidence of CVD was significantly lower in the intensive treatment compared with the routine care (control) group. There was no difference in the mortality experience of the two groups.

We previously showed that the training of general practitioners in intensive treatment of diabetes in the ADDITION-Denmark study had no spillover effect on progression to diabetes in individuals with impaired fasting glucose or impaired glucose tolerance [7]. There are very few other studies examining the spillover effect of a trial intervention in general practice with which to compare our results. In general, interventions targeting healthcare professionals seem to be beneficial only if baseline HbA_1c_ control is poor [8, 9]. Our finding of reduced rates of CVD among individuals with normoglycaemia may therefore be considered somewhat surprising. However, as individuals who have a positive diabetes risk score are at high risk of CVD and mortality whether or not subsequent testing shows them to have diabetes [10], this is a welcome observation. While we did not find a difference in the rates of redeemed medication between groups, we hypothesise that training and support of general practitioners in the intensive treatment arm of the trial may have improved the management of lifestyle behaviour, and maybe medication adherence, among patients identified at high risk but without diabetes on biochemical testing. One-third of screen-detected individuals with diabetes in ADDITION-Denmark reported that they had stopped smoking at the 5 year follow-up. Furthermore, this whole cohort lost an average of 2 kg in weight [2]. Similar behavioural responses among individuals identified at high CVD risk may provide a potential mechanism for the observed CVD risk reduction alongside prescribed treatment.

Overall, there was evidence of suboptimal routine treatment among individuals who screened positive for high risk of diabetes but were normoglycaemic upon biochemical testing in ADDITION-Denmark. Under routine care, general practitioners were advised to treat individuals at high CVD risk e.g. with a heart SCORE ≥5. However, even among people with a heart SCORE ≥ 10, the medication data show that less than 22% of individuals were on lipid-lowering treatment in 2005 and less than 42% in 2010. All participants in this group should have been prescribed lipid-lowering drugs according to national guidelines [5]. As well as evidence of ‘undertreatment’, there also appeared to be a considerable delay in starting treatment among this high-risk population.

While our intervention was associated with a small absolute risk reduction for individuals with normoglycaemia in the entire cohort, there was a clinically meaningful risk reduction among individuals at highest CVD risk (heart SCORE ≥10). We suggest that the reduction in cardiovascular events might have been greater if general practitioners were convinced to treat according to guidelines for cardiovascular risk management.

In the current study, the Danish registry system allowed us to investigate the long-term experience of individuals found to have normal glucose tolerance following screening in ADDITION-Denmark practices between 2001 and 2011. Trial groups were well balanced for patient level characteristics at baseline. Outcome ascertainment was very robust. The Danish National Death Registry estimates 100% coverage of mortality based on death certificates, while the National Patient Registry includes 99.4% of discharges from Danish hospitals. The vast majority of participants were white European, the main ethnic group in Denmark, which limits generalisability to other settings. It would have been interesting to examine trends in lifestyle factors, such as smoking, diet and physical activity, which might have accounted for some of the difference in CVD rates we observed, but data were not available for this cohort.

In conclusion, training of general practitioners to provide target-driven intensive management of blood glucose levels and other cardiovascular risk factors showed some evidence of a spillover effect on the risk of CVD among the normoglycaemic population. The effect was particularly pronounced among those at highest risk of CVD.

## Abbreviations

CVD: Cardiovascular disease
RBG: Random blood glucose
